# Integration of Ecological Momentary Assessment and Intervention With Wearable-Based Digital Phenotypes to Treat Anxiety in Young Adults: Mixed Methods Experimental Study

**DOI:** 10.2196/88919

**Published:** 2026-10-08

**Authors:** Hyunsil Song, Yoobin Choi, Jinho Park, Hyunchan Hwang, Jinwoo Kim, Chanmi Park

**Affiliations:** 1 HAII Corp Jongno-gu Republic of Korea; 2 Yonsei University Seodaemun-gu, Seoul Republic of Korea; 3 Medical IT Convergence Research Center Korea Electronics Technology Institute Seongnam-si Republic of Korea; 4 Chung-Ang University Hospital Dongjak-gu, Seoul Republic of Korea

**Keywords:** mobile health, anxiety disorder, digital phenotypes, ecological momentary assessment, ecological momentary intervention, acceptance and commitment therapy, wearable data

## Abstract

**Background:**

Young adults report high levels of anxiety, yet their access to timely treatment remains limited. Mobile-based digital therapeutics, particularly ecological momentary assessment and intervention (EMA/EMI), offer a promising approach to deliver context-responsive care. The integration of wearable-derived digital phenotypes with digital therapeutics may enhance the personalization of feedback in such interventions, but empirical evidence from real-world settings remains scarce.

**Objective:**

This study examined the real-world applicability and effectiveness of an EMA/EMI-based digital therapeutic, ANZEILAX-Green (HAII Corp), for use in anxiety reduction in young adults and investigated how personalized feedback generated by combining EMA/EMI data with wearable-based digital phenotypes ameliorates symptoms and promotes self-management and user engagement.

**Methods:**

An 8-week exploratory experimental study was conducted with young adults aged 19-39 years. Participants used a smartphone app in which EMA/EMI modules had been integrated with a smartwatch that continuously collected digital phenotyping data, including heart rate, sleep, and activity. Psychological outcomes were assessed using the Generalized Anxiety Disorder 7-item scale (GAD-7) and Patient Health Questionnaire-9 (PHQ-9) at baseline and weeks 2, 4, 6, and 8, and the Rosenberg Self-Esteem Scale (RSES) at baseline and week 8. Quantitative changes were analyzed using paired-samples *t* tests and repeated-measures ANOVA, and qualitative interviews were thematically analyzed to identify user experience mechanisms and design implications.

**Results:**

In all, 29 participants were included in the final analysis. Significant improvements were observed in their GAD-7 (mean difference –4.79, 95% CI –6.56 to –3.02; Cohen d=1.03; *P*<.001), PHQ-9 (mean difference –3.86, 95% CI –5.79 to –1.93; Cohen d=0.76; *P*<.001), and RSES scores (mean difference +5.21, 95% CI 3.47 to 6.95; Cohen d=1.14; *P*<.001). Consistent improvements in GAD-7 scores were observed across the assessment points, with statistically significant reductions from week 4 onward. Participants demonstrated high adherence, completing 80% of the EMA/EMI prompts. The digital phenotype showed trends of increased deep sleep and reduced mobility, and sleep indicators were significantly correlated with depressive symptoms. Analysis of the interview findings revealed 5 mechanisms: enhanced self-awareness, data-driven reflection, behavioral change, intrinsic motivation, and extension of treatment into daily life.

**Conclusions:**

EMA/EMI-based digital therapeutics integrated with wearable-derived digital phenotypes are feasible and acceptable for use by young adults and were associated with within-group reductions in anxiety, suggesting acceptable efficacy, though this warrants confirmation in a controlled trial. The combination of real-time self-reported assessments of emotional states and passive physiological monitoring enables personalized feedback suited to users’ emotional and physiological states. These findings offer empirical and design insights for automated, personalized mental health intervention systems.

## Introduction

Anxiety disorders are a major global mental health concern that affect an estimated 301.39 million people worldwide, accounting for 28.68 million disability-adjusted life years in 2019. Their burden has increased substantially since the COVID-19 pandemic [1,2]. The burden of anxiety disorders is especially pronounced in young adulthood, with the highest prevalence and disability burden observed among individuals aged 20-24 years [3-5]. This age group is especially vulnerable to anxiety due to the pressure of such developmental tasks as academic pursuits, career planning, identity formation, financial independence, and complex social relationships. Thus, timely and accessible early intervention is crucial to reduce delays in care and mitigate the risk of persistent symptoms and long-term functional impairment [6-8], yet treatment use remains remarkably low due to a variety of barriers, including mental health stigma, limited mental health literacy, negative attitudes toward treatment, accessibility constraints, and financial burden [9-11].

Traditional assessments and treatments for anxiety primarily rely upon retrospective self-reports, which fail to capture the dynamic fluctuations of anxiety, which may change rapidly depending on the situation and are vulnerable to recall bias [12-14]. This may cause the aggravation of momentary symptoms and contextual triggers to be overlooked, undermining the accuracy of assessment and the design of personalized intervention.

As a result, digital therapeutics (DTx) have emerged as a promising adjunctive approach to deliver accessible and scalable mental health interventions [15-17]. DTx provide evidence-based therapy at any time and place, decrease financial burdens, and facilitate self-management in daily life [16,18,19]. Given the high engagement with digital technologies among the young, mobile-based DTx may usefully complement traditional mental health care by offering accessible, on-demand therapeutic content, facilitating symptom monitoring, and providing tailored feedback in daily-life contexts [4,20,21]. Numerous studies have reported that mobile-based interventions exerted a positive effect on anxiety by alleviating symptoms [22-24], and DTx hold the potential to resolve issues of cost and accessibility while enhancing users’ self-management abilities [25-27].

Of the variety of DTx approaches currently available, ecological momentary assessment (EMA) is particularly promising, as it repetitively samples users’ responses in their natural daily environments, thereby precisely capturing the momentary fluctuations and context-specific triggers of anxiety [12,14,28]. The implementation of EMA on mobile-based platforms can facilitate the real-time capture of self-reported psychological states in everyday contexts, support time-stamped data collection, and achieve generally good participant compliance [14,29]. Ecological momentary intervention (EMI) uses data derived through EMA to deliver interventions in real time within the context of daily life [30-32], thereby providing therapy right when symptoms occur or when individuals are most receptive to the therapy content [30-33]. By delivering support in daily-life contexts, EMI may help individuals practice and apply therapeutic skills outside clinical settings, which may support the generalization and maintenance of treatment gains [32,34,35]. Together, EMA and EMI represent a promising approach for overcoming the limitations of traditional anxiety treatments [36,37].

Digital phenotype refers to the continuous traces that individuals generate in their everyday interactions with digital technologies, which may reflect health- and illness-related characteristics in real-world contexts [38,39]. EMA/EMI systems may be further enhanced through their integration with wearable-based digital phenotyping by enabling continuous, passive, and context-sensitive monitoring for more personalized and timely interventions [39-42]. Comprising behavioral, physiological, and environmental data collected via smartphones or wearable devices, a digital phenotype represents a person’s real-time digital traces and enables the quantitative characterization of moment-to-moment changes in an individual’s state [39,42-45]. It can thus capture natural variations in physiological states through indicators such as heart rate, sleep patterns, and activity levels that may signal fluctuations in mental health and stress-related states, although the capacity of wearables to reliably infer such states in real-world settings remains limited [43,46-48].

Wearable-derived digital phenotypes complement conventional EMA by allowing an individual’s emotional and physiological states to be captured separately from repeated self-reports. For example, changes in heart rate variability (HRV), activity levels, or sleep patterns may indicate anxiety-related states in daily life [49,50], and when these physiological patterns are examined together with emotional or situational information reported through EMA, they can provide a more comprehensive understanding of an individual’s state [45,50-53].

Additionally, subjective emotional reports and wearable-derived physiological signals may provide complementary rather than redundant information about anxiety-related states [50,54], supporting a more contextualized understanding of individuals’ moment-to-moment experiences and enhancing self-reflection in daily life [52,53,55]. Feedback integrating these subjective and physiological data not only provides information but also serves as a therapeutic tool to boost self-efficacy and treatment adherence [56].

Integrating digital phenotyping into EMA/EMI systems enables more refined mental health assessments by considering subjective experiences and objective physiological indicators together. Such an integrated approach provides a robust foundation for timely personalized interventions grounded in an individual’s physiological and behavioral context, ultimately supporting a more accurate understanding of complex mental health states that cannot be captured with data from a single source.

Despite the promise offered by combining subjective self-reports and objective physiological data in this way, research integrating these approaches remains limited, particularly that comprehensively examining the relationships between EMA/EMI data and physiological signals [57]. Accordingly, further investigation is needed regarding the potential contributions of combined digital-phenotype and EMA/EMI systems to the design of automated intervention systems.

Accordingly, we conducted a study using “ANZEILAX” (HAII Corp; a blend of “anxiety” and “relax,” reflecting the app’s aim of relieving anxiety), a digital therapeutic whose efficacy in treating anxiety disorders has been empirically validated [58]. A randomized controlled trial demonstrated that ANZEILAX significantly alleviated symptoms in patients with generalized anxiety disorder when delivered in combination with usual care, including pharmacotherapy [58]. This study integrated EMA and EMI functions into this proven therapeutic system to assess its real-world applicability and potential for the design of automated intervention systems.

This study explored the real-world applicability of EMA/EMI digital interventions for anxiety reduction among young adults and evaluated their effectiveness. Specifically, it investigated the impact of personalized feedback generated by an EMA/EMI system integrated with a wearable-based digital phenotypic data system on anxiety reduction and self-management capabilities. In addition, this study qualitatively examined the associations of users’ experiences with EMA/EMI systems and integrated digital phenotypic feedback with anxiety-related physiological indicators to identify key design features for automated interventions.

Ultimately, this study sought to provide empirical evidence to support the effective implementation of context-responsive digital interventions, and to help advance personalized intervention strategies to improve mental health in young adults.

## Methods

### Study Design

This study was a follow-up to a randomized controlled trial of ANZEILAX, a digital therapeutic designed to alleviate anxiety symptoms in young adults. This 8-week exploratory single-group study examined the real-world applicability of interventions that integrate EMA/EMI-based digital therapeutics with wearable-derived digital phenotypic data and evaluated the operation of intervention routines, user acceptance, and potential changes in clinical outcome measures before a controlled clinical evaluation of the EMA/EMI-based DTx.

This study used the ANZEILAX-Green smartphone app (HAII Corp) with integrated EMA/EMI functions in conjunction with a smartwatch-based tool that collects health data to comprehensively explore real-time emotion assessment, momentary intervention delivery, and changes in behavioral and physiological responses in everyday contexts. The study was conducted from September to November 2025. An overview of the study design and procedures is presented in Figure 1.

**Figure 1:**
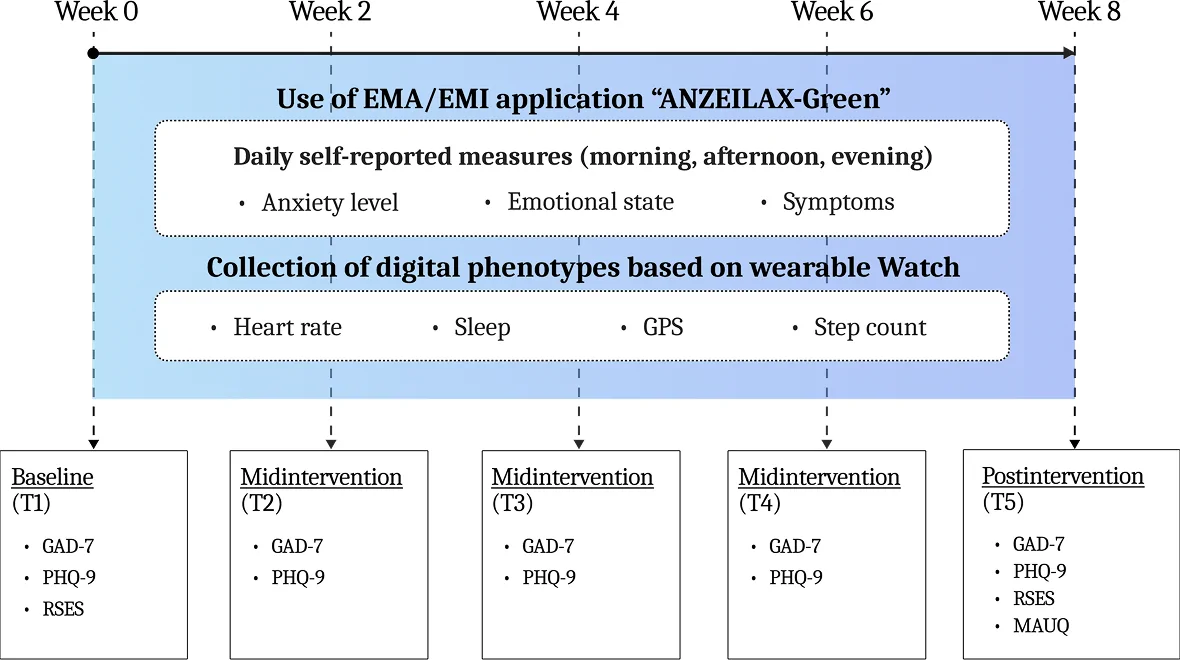
Study design and ecological momentary assessment and intervention data collection flow. Overview of the 8-week study design, interventions, and collected digital phenotypes. EMA/EMI: ecological momentary assessment and ecological momentary intervention; GAD-7: Generalized Anxiety Disorder 7-item scale; MAUQ: mHealth App Usability Questionnaire; PHQ-9: Patient Health Questionnaire-9; RSES: Rosenberg Self-Esteem Scale.

### Ethical Considerations

This study was reviewed and approved by the Institutional Review Board of Yonsei University (IRB number 7001988-202509-HR-2892-03). All participants provided written informed consent after receiving a full explanation of the study purpose and procedures from the research team. Participant confidentiality was maintained throughout the study, with each individual being assigned a numerical code by their order of participation. All collected data were anonymized to ensure participant privacy. Participants who completed the 8-week study were compensated with KRW 130,000 (approximately US $88) for their time and effort, and those who completed the postintervention interview received an additional KRW 20,000 (approximately US $14) as supplementary compensation.

### Participants

The participants were Korean young adults aged between 19 and 39 years, with a Beck Anxiety Inventory (BAI) score of 8 to 25 (indicative of mild-to-moderate anxiety), who could communicate in Korean and use smartphones, and included individuals who had not been prescribed psychiatric medication by a psychiatric department within 30 days before the start of the study or those without changes in medication if they had been taking any. Individuals with current or past psychiatric disorders (including schizophrenia, psychosis, bipolar disorder, and epilepsy), brain injury, cognitive impairment, neurological disorders, intellectual disability, or substance or alcohol use disorders, as well as those who had exhibited suicidal intent, suicidal impulses, or self-harming behavior within the past 6 months, were excluded. High-risk individuals with BAI scores of 26 or higher were also excluded.

### Procedures

Purposive sampling was used to ensure sufficient representation across age groups and anxiety severity levels (mild and moderate). Participants were approached by a member of the research team who had not been involved in quantitative data collection so as to minimize response bias. A total of 30 participants were enrolled after the research team confirmed their eligibility through online recruitment. After providing informed consent, the participants completed baseline assessments covering their sociodemographic characteristics, anxiety, and depression and then downloaded the ANZEILAX-Green app (HAII Corp). They used the app for 8 weeks in combination with a Galaxy Watch 7 to be worn throughout the study period. On the enrollment day, all participants received hands-on onboarding instructing them in the use of the app and the wearable device and were instructed to contact the research team at any time if they encountered technical difficulties.

Participants completed survey evaluations at weeks 2, 4, and 6, followed by a postintervention assessment at week 8, and those who had volunteered were invited to take part in in-depth interviews following the intervention; all 29 participants who had completed the 8-week intervention voluntarily agreed to participate in the interviews. A research team experienced in qualitative interviews conducted semistructured interviews to explore their experiences with the intervention and elicit suggestions for improvement. The key topics included participants’ overall experience with the app, their perceptions of its effectiveness and satisfaction, and any facilitating and hindering factors they had noted. In particular, an interview guide was developed a priori based on the study objectives and reviewed by the research team for content validity, and the following topics were set in advance: (1) overall experience with the ANZEILAX-Green app and wearable device, (2) perceived effectiveness and satisfaction with the intervention, (3) factors facilitating and hindering engagement, (4) perceptions of the personalized weekly feedback reports, and (5) suggestions for improvement.

All interviews were conducted individually via video call after the intervention period had ended and were audio-recorded with participants’ prior written consent. Interviews lasted approximately 30-60 minutes. The interviewer maintained a reflexive stance throughout, bracketing their assumptions about the intervention and remaining open to participants’ perspectives. The audio recordings were transcribed verbatim by a research assistant and reviewed for accuracy before analysis. Transcripts were not returned to participants to be checked at this stage. However, participant quotations representative of each theme were verified against the original transcripts to ensure accuracy.

### Intervention

#### Personalized Emotion Regulation App Based on EMA/EMI Systems

The intervention is based on the mobile app ANZEILAX, which received approval from the Korea Food and Drug Safety Administration in April 2025. ANZEILAX incorporates a variety of therapeutic modules, including a self-talk program based on acceptance and commitment therapy (ACT), to support users’ psychological flexibility. EMA- and EMI-based functions were integrated into ANZEILAX to evaluate users’ emotional states in real time and automatically provide tailored interventions, enabling more dynamic and personalized intervention delivery than the original version. This integrated version, named ANZEILAX-Green, was developed for the Android platform and launched in 2025 by HAII Corp.

In this study, participants were primarily instructed to use the EMA/EMI functions, while the other therapeutic modules in ANZEILAX were available for optional use. The participants completed EMA questionnaires regarding their anxiety and emotional states through the smartphone app multiple times per day at self-scheduled notification times in the morning, afternoon, and evening. They were required to respond to at least 3 assessment prompts per day over the 8-week period. The questionnaire consisted of items assessing anxiety (1-7 points), positive and negative emotions (each 1-7 points), and symptoms (10 items covering physical, cognitive, and behavioral domains). Positive and negative emotions were assessed using an adapted version of the International Positive and Negative Affect Schedule Short Form (I-PANAS-SF) [59].

The app analyzes users’ EMA responses (anxiety, positive and negative emotions, and symptom scores) in real time and supports the automated delivery of EMI content based on ACT principles that induce immediate emotional relief through adaptive delivery based on real-time conditions. The EMA/EMI workflow is as follows: (1) upon EMA input of a user’s condition, the system calculates priorities, and (2) automatically selects the intervention type (audio meditation, image, or text) appropriate to the condition and immediately provides EMI content.

The app was designed to strike a balance between automation and personalization. It functions as a state-responsive digital treatment model in which interventions are determined based on a user’s actual emotional state, instead of unilateral push interventions. Users thereby learn to recognize their emotional changes in real time and immediately experience the results of interventions, thereby cultivating their emotional self-regulation skills. Detailed screenshots of the app interface are presented in Figure 2.

**Figure 2:**
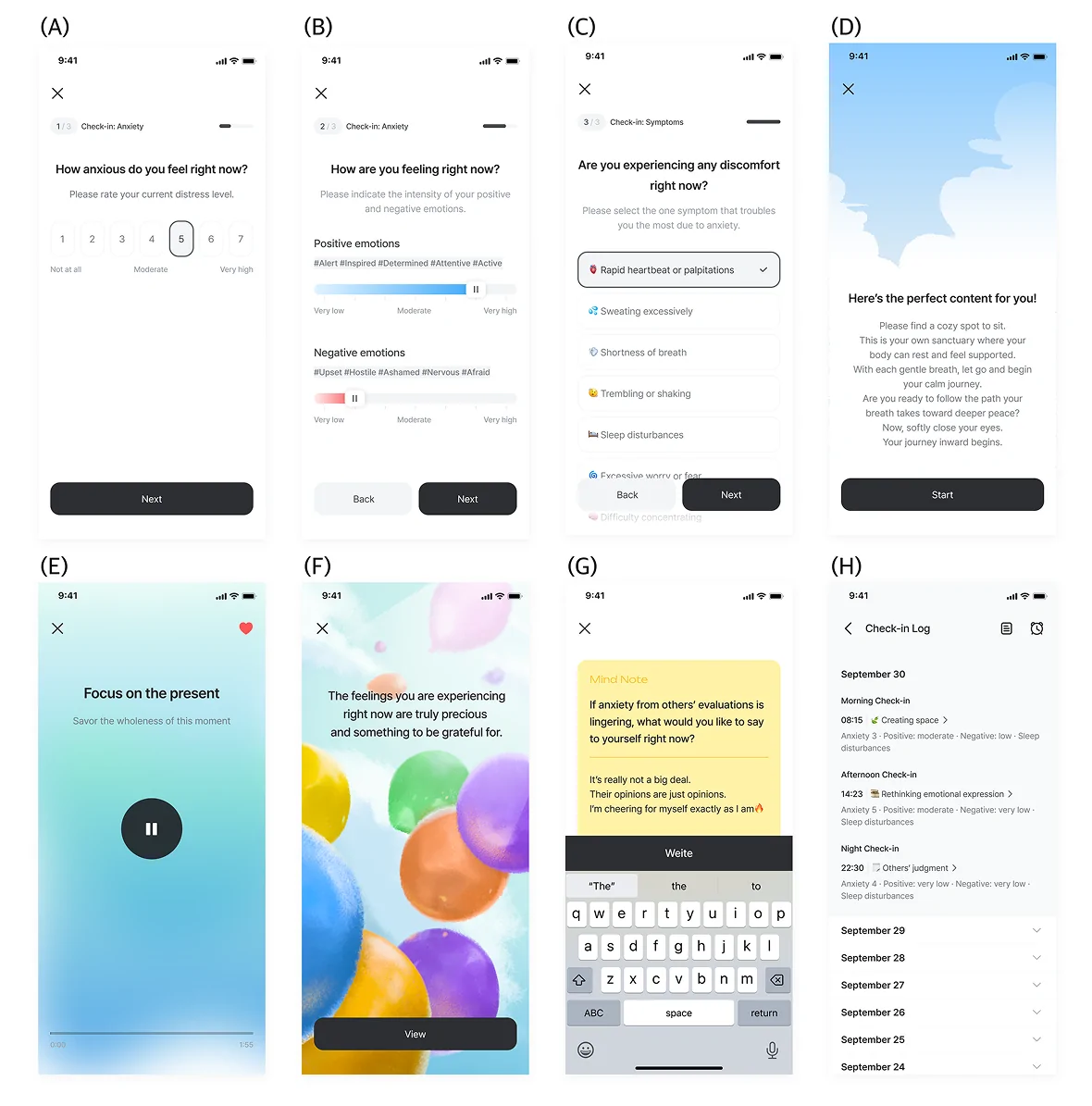
ANZEILAX-Green app. From left to right, top to bottom: (A-C) ecological momentary assessment (EMA) screens for assessing anxiety, positive emotions, negative emotions, and symptoms. (D) Introduction and entry screen for personalized ecological momentary intervention (EMI) content. (E) Audio-type EMI featuring guided breathing meditation. (F) EMI with images and scripted guidance. (G) EMI encouraging reflection by reading a column and writing thoughts. (H) Tracking screen for EMA and EMI task completion records.

#### Digital Phenotype Collection App

Digital phenotypes, including heart rate, sleep patterns, and activity levels, were automatically collected in the background through Samsung Health integrated into the Android platform. Physiological data were captured via synchronization between the smartwatch and smartphone. Researchers have examined the validity of consumer smartwatch sensors for heart rate and sleep-stage estimation [60,61]. Nonetheless, such devices remain less accurate than polysomnography for sleep-stage classification, which was taken into consideration in data interpretation.

The digital phenotype indicators collected in this study reflect physiological and behavioral responses known to be associated with anxiety. Heart rate–related indicators are representative physiological markers of autonomic nervous system activity reported to reflect heightened arousal and autonomic imbalance associated with increased anxiety levels. Location (GPS) data and mobility patterns have been shown to capture social avoidance behaviors related to anxiety objectively [62]. Sleep-related indicators are also closely linked to anxiety, as individuals reporting higher anxiety levels tend to exhibit poorer sleep quality [63]. Finally, activity-related indicators, including step count and movement patterns, have been used as behavioral markers reflecting avoidance behaviors and irregular daily activity associated with anxiety [56]. However, because heart rate is also elevated by physical activity, the heart rate indicators were not interpreted as anxiety-specific markers, and the absence of activity adjustment constitutes a limitation of this approach [64].

The collected data thus included physiological and behavioral indicators strongly associated with anxiety and related mental health conditions and were analyzed alongside quantitative assessment measures over time. Before the analysis, preprocessing procedures, including the removal of missing values and sensor-specific corrections such as segment adjustments, were performed. A detailed list of the collected digital phenotype items is presented in Textbox 1.

**Description of digital phenotypes.**

**Heart**

- Heart rate (HR): average HR collected at 1- to 3-second intervals.

**GPS**

- Latitude: latitude value collected every 5 minutes.
- Longitude: longitude value collected every 5 minutes.

**Sleep**

- Light sleep duration: duration of light sleep per sleep event.
- Total sleep duration: total sleep duration per sleep event.
- Rapid eye movement duration: duration of rapid eye movement sleep per sleep event.
- Deep sleep duration: duration of deep sleep per sleep event.

**Steps**

- Step count: number of steps taken across a 5-minute interval.

#### Personalized Weekly Feedback Report

While the participants engaged with the ANZEILAX-Green, physiological data such as heart rate, sleep, and activity levels were simultaneously collected via the wearable device as digital phenotype data and reported weekly as objective biometrics. The weekly report summarized app usage, key usage patterns, daily anxiety scores, reported emotions and symptoms, activity levels, heart rate, and sleep metrics and provided a personalized summary with recommended activities. These biometrics facilitated the translation of abstract sensations into concrete, interpretable indicators, thus fostering a clear recognition of one’s state. The long-term patterns could help users identify personal triggers and protective factors, while regular data reviews could promote sustained attention and reminder effects, which can prompt behavioral change. Feedback based on objective numbers can be an effective therapeutic tool to enhance self-efficacy and treatment adherence (Figure 3).

**Figure 3:**
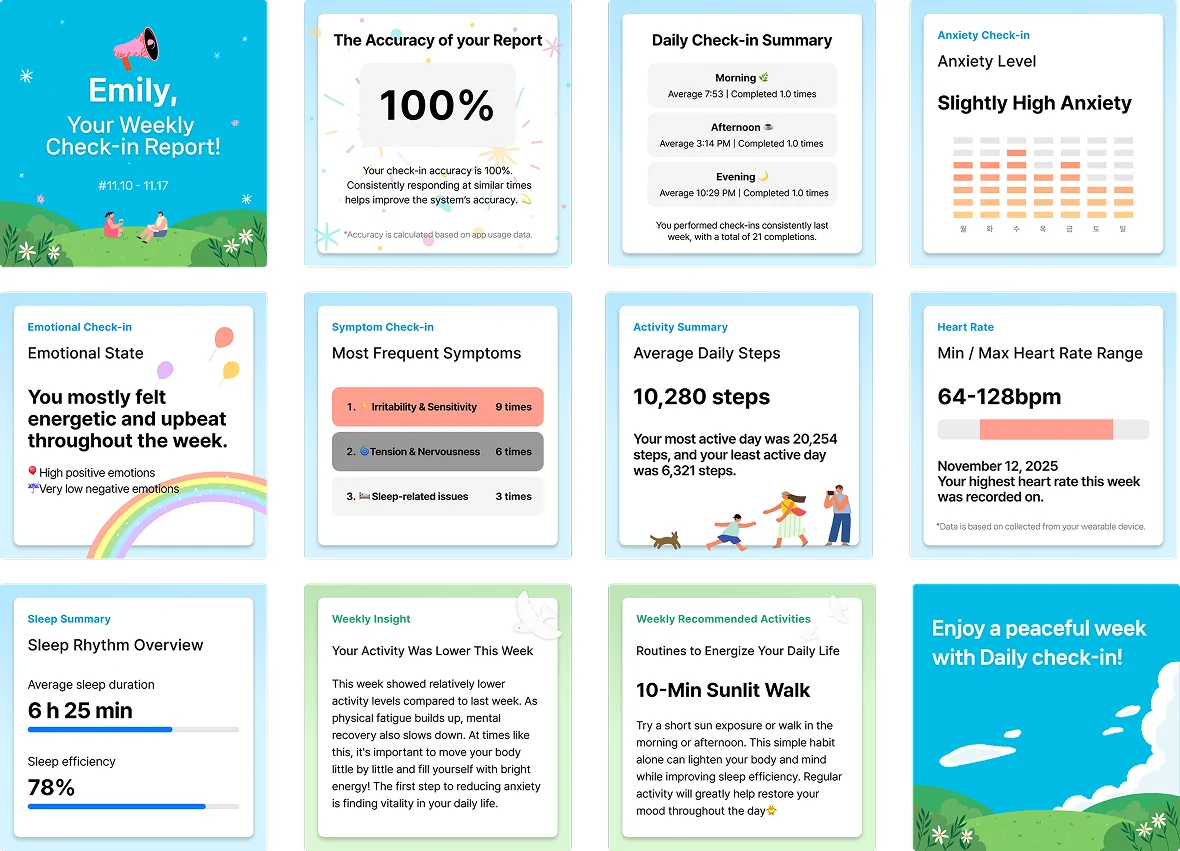
ANZEILAX-Green weekly report for participants.

### Measures

This study used the following measures. The Generalized Anxiety Disorder 7-item scale (GAD-7) was administered to assess anxiety symptom relief [65] and the Patient Health Questionnaire-9 (PHQ-9) to evaluate depressive symptoms, which frequently co-occur with and are closely associated with anxiety [66]. Self-esteem, a psychological construct associated with anxiety levels, was assessed using the Rosenberg Self-Esteem Scale (RSES) [67]. Finally, app usability was evaluated using the mHealth App Usability Questionnaire (MAUQ) [68].

### Statistical Analysis

Analyses were conducted using SPSS version 29.0 statistical software (IBM Corp). Outcome measures were analyzed using repeated-measures ANOVA to examine changes over time. When the assumption of sphericity was violated, the Greenhouse-Geisser correction was applied, and pairwise differences across time were examined using post hoc comparisons with Bonferroni adjustment. Associations between digital phenotype indicators and clinical outcome measures were examined using ordinary least squares (OLS) regression implemented in Python (version 3.12; Python Software Foundation).

### Qualitative Data Analysis

The qualitative data from the semistructured interviews were analyzed independently by 2 researchers using reflexive thematic analysis following the 6-phase framework of Braun and Clarke [69]. This approach was selected for its flexibility and suitability for exploratory research aimed at identifying experiential patterns across participants. The analysis proceeded inductively without a predetermined coding framework, allowing themes to emerge from the data.

The six phases were as follows: (1) familiarization, in which both researchers read and reread the transcripts and recorded initial impressions; (2) systematic coding, in which meaningful units of data were assigned descriptive codes; (3) searching for themes, in which codes were collated into candidate themes; (4) reviewing themes, in which the coherence and distinctiveness of the themes were evaluated in relation to the full dataset; (5) defining and naming themes, in which each theme was clearly delineated and given a descriptive name; and (6) writing up, in which themes were reported with supporting participant quotations.

To enhance the rigor and dependability of the analysis, each researcher coded the transcripts independently, and discrepancies were resolved through iterative discussion until consensus was reached. Initial coding was compared to ensure consistency. Credibility was supported through the use of verbatim participant quotations illustrating each theme and by grounding interpretations in the data. Transferability was addressed through a thick description of the study context, setting, and participant characteristics. Confirmability was maintained by documenting analytic decisions throughout the process. Reflexivity was practiced throughout the analysis, with both researchers reflecting on how their professional backgrounds and prior knowledge of the intervention might have shaped their interpretation of the data. All qualitative data were managed and organized using a structured coding matrix in Microsoft Excel.

## Results

### Demographic Information

A total of 61 individuals were screened for eligibility in this study. Of these, 31 were excluded because their BAI scores fell outside the range of 8-25. The other 30 participants met all the inclusion criteria and were enrolled.

Table 1 provides an overview of the demographics and baseline clinical characteristics. The mean age was 29.83 (SD 5.81) years, with participants evenly distributed between the 19-29 (15, 50%) and 30-39 years age groups (15, 50%). The majority of the participants were women (21/30, 70%), and the remaining 9/30 (30%) were men. Most participants had completed an undergraduate degree or higher, and about half were employed. The reported duration of anxiety symptoms varied, most commonly falling in the range of 1-3 years. The mean BAI score, which was used for screening, was 15.03 (SD 4.12), with 17/30 (57%) participants classified as having mild anxiety and 13/30 (43%) moderate anxiety.

Baseline outcome scores indicated moderate levels of anxiety and depressive symptoms, with mean scores of 10.70 (SD 2.85) on the GAD-7 and 10.50 (SD 4.47) on the PHQ-9. The mean baseline score on the RSES was 29.13 (SD 7.22). Based on the baseline GAD-7 scores, 10/30 (33%) participants were classified as having mild, 17/30 (57%) moderate, and 3/30 (10%) severe anxiety. Based on their PHQ-9 scores, 3/30 (10%) had none-to-minimal, 11/30 (37%) mild, 9/30 (30%) moderate, and 7/30 (23%) moderately severe depressive symptoms, with none classified as severe (Table 1).

**Table 1:** Demographic and clinical characteristics of the participants (N=30).

Characteristics			Values
Age, mean (SD)			29.83 (5.81)
**Age group (years),** **n** **(%)**			
	19-29	15 (50)	
	30-39	15 (50)	
**Sex,** **n** **(%)**			
	Male	9 (30)	
	Female	21 (70)	
**Education,** **n** **(%)**			
	Doctoral degree	1 (3)	
	Master’s degree	3 (10)	
	Undergraduate degree	18 (60)	
	High school degree	8 (27)	
**Work status,** **n** **(%)**			
	Employed	15 (50)	
	Not employed	10 (33)	
	Student	5 (17)	
**History of anxiety symptoms,** **n** **(%)**			
	≤3 months	5 (17)	
	6 months to 1 year	5 (17)	
	1-3 years	11 (37)	
	3-5 years	2 (7)	
	>5 years	7 (23)	
BAI^a^, mean (SD)			15.03 (4.12)
**BAI severity,** **n** **(%)**			
	Mild anxiety	17 (57)	
	Moderate anxiety	13 (43)	
**GAD-7^b^ severity, n (%)**			
	Mild (5-9)	10 (33)	
	Moderate (10-14)	17 (57)	
	Severe (15-21)	3 (10)	
**PHQ-9^c^ severity, n (%)**			
	None-to-minimal (0-4)	3 (10)	
	Mild (5-9)	11 (37)	
	Moderate (10-14)	9 (30)	
	Moderately severe (15-19)	7 (23)	
	Severe (20-27)	0 (0)	
**Outcomes, means (SD)**			
	Baseline GAD-7	10.70 (2.85)	
	Baseline PHQ-9	10.50 (4.47)	
	Baseline RSES^d^	29.13 (7.22)	

^a^BAI: Beck Anxiety Inventory.

^b^GAD-7: Generalized Anxiety Disorder 7-item scale.

^c^PHQ-9: Patient Health Questionnaire-9.

^d^RSES: Rosenberg Self-Esteem Scale.

### Intervention Effects on Psychological Outcomes

A total of 29 participants were included in the final analysis. One participant was excluded because they did not respond to contact after the baseline assessment.

The main findings are as follows. Compared with the baseline, the GAD-7 and PHQ-9 scores showed significant within-group reductions in anxiety (GAD-7) and depression (PHQ-9), and a significant increase was found in self-esteem (RSES) scores. Please note that the following are within-group changes from a single-arm design without a control group. The mean GAD-7 score decreased from 10.66 (SD 2.89) at baseline to 5.86 (SD 4.34) at the completion of the intervention, with an average change of –4.79 points (95% CI –6.56 to –3.02; Cohen *d*=1.03; *P*<.001). The mean PHQ-9 score decreased from 10.31 (SD 4.43) to 6.45 (SD 4.99) over the same period, with an average change of –3.86 points (95% CI –5.79 to –1.93; Cohen *d*=0.76; *P*<.001), while the mean RSES score increased from 29.34 (SD 7.25) to 34.55 (SD 7.31) over this period, with an average change of 5.21 points (95% CI 3.47 to 6.95; Cohen *d*=1.14; *P*<.001), indicating a significant improvement in self-esteem over the course of the intervention (Table 2).

**Table 2:** Impact of changes in outcomes from baseline to postintervention (N=29).

Outcomes	Baseline (week 0), mean (SD)	Postintervention (week 8), mean (SD)	Δ Mean (95% CI)	Effect size (Cohen *d*)	*P* value
GAD-7^a^	10.66 (2.89)	5.86 (4.34)	–4.79 (–6.56 to –3.02)	1.03	<.001
PHQ-9^b^	10.31 (4.43)	6.45 (4.99)	–3.86 (–5.79 to –1.93)	0.76	<.001
RSES^c^	29.34 (7.25)	34.55 (7.31)	5.21 (3.47 to 6.95)	1.14	<.001

^a^GAD-7: Generalized Anxiety Disorder 7-item scale.

^b^PHQ-9: Patient Health Questionnaire-9.

^c^RSES: Rosenberg Self-Esteem Scale.

The changes over time also exhibited consistent improvement, as reflected by ongoing reductions in the symptom severity scores. Gradual improvements from the midpoint assessment were noted for anxiety and depression, with a significant reduction observed at week 4 during the early phase of the intervention. The downward trend continued steadily through weeks 6 and 8, with the most pronounced improvement observed postintervention. Anxiety symptoms, measured by GAD-7, showed a steady and sustained decrease from week 4 onward, while depressive symptoms, measured by PHQ-9, followed a similar overall trajectory. Although the PHQ-9 scores in week 8 were numerically slightly higher than in week 6 (not formally tested), they remained significantly lower than baseline at postintervention (Table 3; Figure 4).

**Table 3:** Changes in GAD-7a and PHQ-9b scores over the intervention period (N=29).

Outcomes			Mean (SD)		Δ Mean (95% CI)		*P* value versus baseline
**GAD-7**							
	Midintervention (week 2)	8.07 (4.22)		−2.59 (−5.20 to 0.03)		.05	
	Midintervention (week 4)	7.48 (4.32)		−3.17 (−5.74 to −0.60)		.008	
	Midintervention (week 6)	6.10 (3.90)		−4.55 (−6.83 to −2.27)		<.001	
	Postintervention (week 8)	5.86 (4.34)		−4.79 (−7.43 to −2.16)		<.001	
**PHQ-9**							
	Midintervention (week 2)	8.07 (5.05)		−2.24 (−4.95 to 0.46)		.18	
	Midintervention (week 4)	7.52 (5.27)		−2.79 (−5.45 to −0.14)		.03	
	Midintervention (week 6)	6.41 (3.94)		−3.90 (−6.23 to −1.56)		<.001	
	Postintervention (week 8)	6.45 (4.99)		−3.86 (−6.73 to −0.99)		.003	

^a^GAD-7: Generalized Anxiety Disorder 7-item scale.

^b^PHQ-9: Patient Health Questionnaire-9.

**Figure 4:**
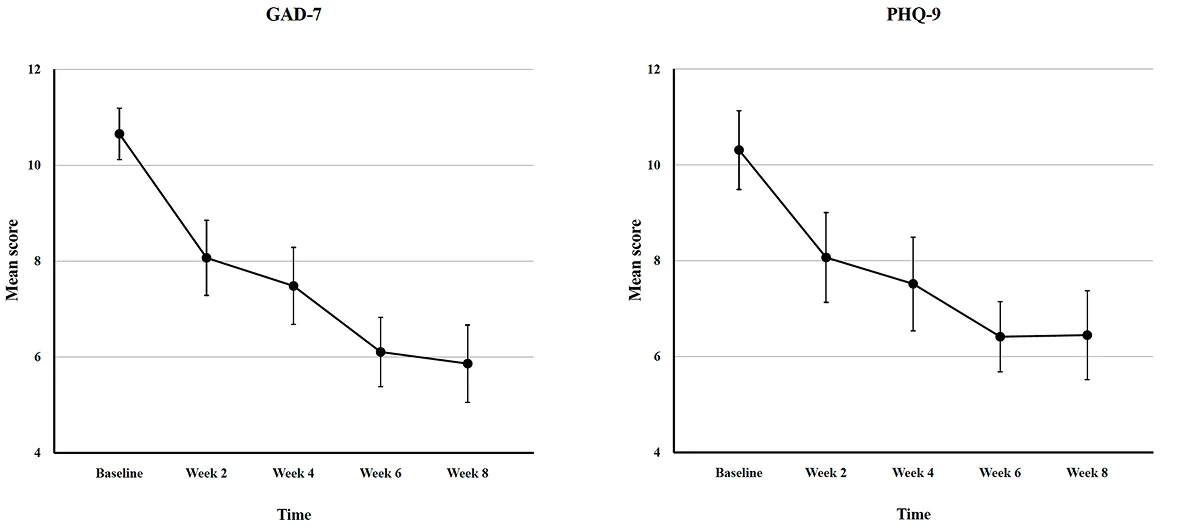
Mean GAD-7 (Generalized Anxiety Disorder 7-item scale) and PHQ-9 (Patient Health Questionnaire-9) scores at baseline and weeks 2, 4, 6, and 8 during the 8-week intervention.

These patterns of change suggest consistent improvements in symptoms throughout the intervention, with early changes significantly contributing to the overall effect. The effect sizes and temporal change patterns observed in this study are consistent with those reported in previous ANZEILAX clinical studies [58]. In particular, rapid responses and sustained effects on anxiety symptoms may be of clinical interest, although this requires confirmation by research using controlled designs.

### Adherence and Usability

Adherence was calculated based on the protocol requirement of 3 daily EMA/EMI completions over the 8-week intervention period. The participants maintained an average compliance frequency of 80%, thus adhering to the EMA/EMI protocols at a consistently high level.

The heatmap presented in Figure 5 displays the adherence patterns of the 29 participants along the vertical axis over 56 days. Colors closer to green indicate higher adherence, with 1.0 representing full compliance, whereas those closer to gray indicate lower adherence. Blue indicates additional EMA/EMI completions beyond the prescribed 3 daily completions.

**Figure 5:**
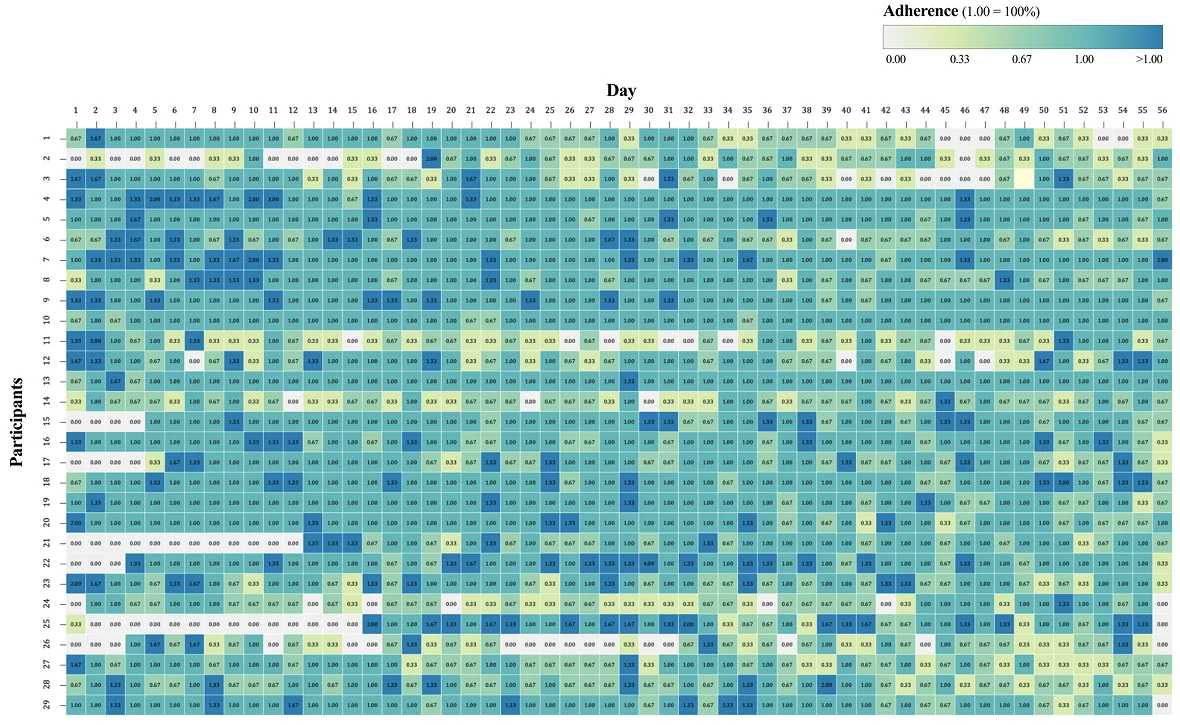
Daily ecological momentary assessment and ecological momentary intervention completion patterns among participants during the 8-week intervention.

As the heatmap illustrates, most participants consistently completed 2 to 3 EMA/EMI reports per day throughout the study, thus maintaining stable use patterns. The average adherence to EMA/EMI remained stable throughout the study period, with no sharp declines at any specific time points or weeks, despite variations in completion frequency among participants.

After the 8-week intervention period, participants completed the MAUQ to assess the usability of the app. The MAUQ results showed an average score of 5.03 (SD 1.32) on a 7-point Likert scale. Among the subscales, ease of use and satisfaction scored the highest at 5.28 (SD 1.28), followed by information organization at 4.98 (SD 1.31) and usefulness at 4.84 (SD 1.36). These results indicate that participants rated the overall usability of the app as favorable, while also making suggestions to further optimize the user experience.

### Digital Phenotypes

In this study, several digital phenotypes, including heart rate, GPS-derived mobility indicators, sleep patterns, and activity levels measured by step count, were collected and analyzed to comprehensively detect changes in participants’ mental health status. The procedures for preprocessing and analysis were applied based on the characteristics of each type of data.

Heart rate data were categorized into 3 ranges: high, ≥100 bpm; medium, 60 bpm to <100 bpm; and low, <60 bpm. The total distance and movement radius were calculated from the GPS latitude and longitude information and normalized from 0 to 1 for comparison between individuals.

To analyze behavioral patterns by time of day, the total distance was classified into daytime and nighttime intervals, with 8 PM as the cutoff. Sleep data were analyzed using Samsung Health’s 4-stage classification, including awake, light, REM, and deep, based on sleep records from 6 PM of the preceding day to noon of the following day. Step count data were also normalized from 0 to 1, as with the GPS-derived indicators.

Weekly averages were calculated for data analysis, and trends over the 8-week period were examined using the ordinary least squares method. Statistically significant changes were observed in several physiological and behavioral indicators. The proportion of measurements within the low heart rate range significantly increased over time (β=0.008, *P*=.02), while GPS-based movement data exhibited a significant decrease in distance over the 8-week period (β=–0.3029, *P*=.03). Among sleep-related indicators, deep sleep duration showed a significant increase (β=0.2624, *P*=.02), whereas light sleep duration showed a decreasing trend. Although changes in step count did not reach statistical significance, an upward trend was observed (β=0.1475, *P*=.11). These wearable-derived indicators were not adjusted for physical activity. In addition, heart rate ranges were defined using fixed rather than individualized thresholds, and consumer-wearable sleep-stage estimates are less accurate than polysomnography. These exploratory findings should thus be interpreted with caution.

Analyses of the correlations between digital phenotyping indicators and clinical measures revealed several significant associations. A significant correlation was identified between PHQ-9 scores and deep sleep duration (*r*=0.655, *P*=.04), indicating an association between depressive symptom severity and deep sleep characteristics. In addition, low heart-rate indicators showed significant positive correlations with both anxiety and depressive symptom severity, as measured by GAD-7 (*r*=0.578) and PHQ-9 (*r*=0.836).

Exploratory analyses found that several digital phenotyping indicators showed concurrent changes during the intervention period. Given the small sample and wide CIs, these associations should be interpreted cautiously (Table 4; Figure 6).

**Table 4:** Correlation analysis between digital phenotypes and GAD-7^a^ and PHQ-9^b^ scores.

Digital phenotype indicator			β (trend estimate)		SE		*P* value		Correlation with GAD-7 (*r*)		Correlation with PHQ-9 (*r*)
**Heart rate**											
	High	–0.0104		0.0079		0.1909		—^c^		—	
	Medium	0.0024		0.0062		0.7042		—		—	
	Low	0.008		0.0033		0.02		0.578^d^		0.836^d^	
**GPS**											
	Total distance	–0.3029		0.1361		0.03		−0.004		−0.222	
	Movement radius	–0.0747		0.1900		0.6942		—		—	
	Daytime distance	–0.0015		0.0468		0.9745		—		—	
	Nighttime distance	0.0550		0.0402		0.1713		—		—	
**Sleep**											
	Awake	0.0180		0.1979		0.9276		—		—	
	Light sleep	–0.2553		0.1412		0.0707		—		—	
	REM^e^ sleep	0.1207		0.1716		0.4815		—		—	
	Deep sleep	0.2624		0.1106		0.02		0.221		0.655^d^	
**Step**											
	Step count	0.1475		0.0935		0.11		—		—	

^a^GAD-7: Generalized Anxiety Disorder 7-item scale.

^b^PHQ-9: Patient Health Questionnaire-9.

^c^Not applicable.

^d^Statistically significant correlation, *P*<.05.

^e^REM: rapid eye movement.

**Figure 6:**
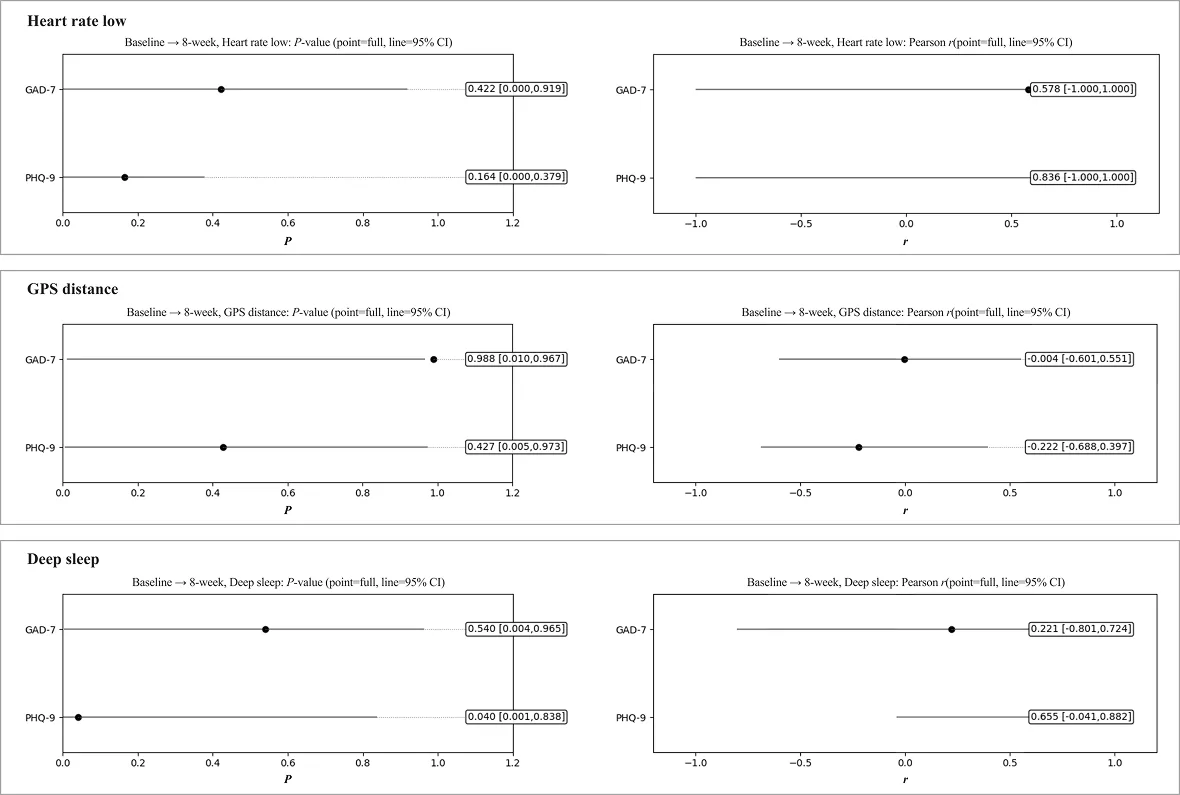
Correlation analysis between digital phenotypes and Generalized Anxiety Disorder 7-item scale (GAD-7) and Patient Health Questionnaire-9 (PHQ-9) scores. Pearson correlation coefficients (r), 95% CIs, and *P* values between digital phenotypes (GPS total distance, deep sleep duration, and lowest heart rate) and GAD-7 and PHQ-9 scores measured at baseline and weeks 2, 4, 6, and 8.

### User Interviews

#### Overview

Poststudy interviews were conducted to explore participants’ experiences with the ANZEILAX-Green app, its integration with wearable devices, and the weekly feedback reports. The interview findings revealed 5 major themes, indicating that the design features of the system enhanced participants’ self-awareness, facilitated data-driven reflection, supported behavioral change, strengthened their intrinsic motivation, and enabled them to apply therapeutic strategies in daily life. At the same time, participants described several challenges, including difficulty in calibrating subjective emotional intensity, the burden of frequent self-monitoring during busy or distressing periods, and heightened awareness of anxiety from repeated self-rating.

#### Theme 1: Enhanced Self-Awareness

Participants reported that the frequent EMA prompts enabled them to notice emotional and physiological changes that had typically gone unnoticed in daily life. Regular check-ins helped structure their self-reflection across different times of day, encouraging a habit of monitoring their internal state.

Being asked several times a day made me pay attention to emotions I would normally ignore... I realized my mood swings were actually more intense than I thought.

P13

Dividing the day into morning, afternoon, and night helped me reflect on each period individually.

P17

Participants also noted increased awareness of the links between physical states and psychological experiences.

Seeing my sleep pattern made me realize why I became irritable and sensitive.

P11

Several participants initially had difficulty in determining the appropriate intensity of their emotions, reflecting the subjective nature of emotional self-assessment. These findings highlight the importance of clear guidance for subjective emotional self-assessment during repeated EMA monitoring.

#### Theme 2: Promoting Data-Driven Reflection

Weekly reports presenting their EMA patterns and digital phenotype data allowed participants to reflect more deeply on their emotional states and interpret their emotional experiences in a clearer, more structured way. Rather than merely displaying data, the reports provided objective cues for reviewing daily patterns and emotional fluctuations that helped the participants identify underlying factors linking their emotions and behaviors and offered useful insights for more intentional self-management.

Seeing everything in one place helped me understand whether the week was good or stressful, and what triggered the fluctuations.

P28

Objective indicators also challenged participants’ inaccurate assumptions about their well-being.

I thought I lacked sleep, but the report showed that fitness was the bigger issue.

P23

These insights helped participants develop more targeted self-management strategies. Several participants reported that the repeated daily check-ins and reflective responses initially felt burdensome, particularly when they were busy or emotionally distressed. These findings suggest that EMA systems should balance repeated self-monitoring with users’ perceived burden [70].

#### Theme 3: Supporting Behavioral Change

Participants stated that personalized EMI content, such as breathing exercises, guided prompts, and reflective messages, motivated immediate action and supported symptom reduction. These interventions were perceived as actionable strategies rather than passive information.

The suggestions in the report made me walk more or adjust my sleep patterns the following week.

P13

Self-monitoring also encouraged intentional shifts in thinking.

When I marked negative emotions (in the app), I tried to reframe the situation more positively.

P03

Brief, affirming messages from EMI provided immediate emotional relief.

Messages like ‘You deserve this happiness’ stayed with me all day and helped me feel calmer.

P05

#### Theme 4: Maintaining Intrinsic Motivation

Participants stated that the routine of checking their status was internalized as a daily habit, helping them sustain their engagement beyond external prompts. Use of the app also reinforced self-efficacy and positive self-regard.

Checking in became a habit. When I didn’t record, something felt missing.

P13

Managing myself boosted my self-esteem and helped me understand myself better.

P25

Personalized and empathetic messages were described as supportive, helping maintain engagement even during periods of distress or positive emotional states.

The messages comforted me when I was anxious and recognized my good moments too.

P21

#### Theme 5: Expansion of Active Treatment

Participants described exploring additional therapeutic modules independently, such as self-talk exercises, meditation, and emotional journaling, through which the treatment effects were extended beyond EMA/EMI. Furthermore, skills acquired through the system transferred into real-life contexts.

I practiced breathing techniques and self-talk almost every day, and it really supported my mental growth.

P23

When I felt anxious offline, I recalled solutions I had learned through the app and applied them naturally.

P29

These findings suggest that an autonomous, flexible environment enhances user agency and broadens the therapeutic impact of the intervention.

## Discussion

### Principal Findings

This study examined the real-world applicability and effectiveness of an EMA/EMI-based digital therapeutic, ANZEILAX-Green, in anxiety reduction among young adults and investigated how the personalized feedback generated by the combined EMA/EMI data and wearable-based digital phenotypes supported symptom improvement, self-management, and user engagement. The results of this 8-week exploratory study indicate that personalized feedback integrating the EMA/EMI system with wearable-based digital phenotype data was associated with improvements in both clinical outcomes and participants’ capacity for autonomous symptom management, although causality cannot be inferred due to the single-arm design of this study.

The intervention was associated with significant improvements in psychological outcomes. GAD-7 scores showed a marked reduction with a large effect size (Cohen d=1.03) and PHQ-9 scores a medium-to-large effect size (Cohen d=0.76), while RSES scores showed a significant increase with a large effect size (Cohen *d*=1.14). The intervention was thus associated with reductions in anxiety symptoms and concurrent improvements in depressive symptoms and self-esteem, suggesting a broader positive impact on overall mental health. GAD-7 scores began to decline from week 2, showed statistically significant reductions from week 4 onward, and continued to improve through postintervention. These effect sizes and temporal change patterns are generally comparable to those reported elsewhere [58].

The magnitude of the observed effects suggests a meaningful therapeutic signal in real-world use. The relatively large effect sizes may in part reflect the single-arm study design, but they also appear to be related to participants’ repeated engagement with the EMA/EMI system in their daily lives. Repeated use of the app likely supported continuous self-monitoring and the timely application of therapeutic strategies. In addition, the study sample primarily consisted of young adults with mild-to-moderate anxiety symptoms and relatively short symptom histories, who might have been more responsive to structured self-management support.

This pattern of early improvement and sustained reduction in symptoms suggests that EMA/EMI-based interventions can help attenuate symptom exacerbation in everyday contexts and support ongoing self-management through the timely delivery of therapeutic content. The EMA/EMI system was designed for frequent use while imposing a low perceived burden through personalized, context-appropriate intervention tasks. This balance might have supported sustained engagement and enabled repeated exposure to therapeutic strategies feasible for use in daily life, which might have contributed to gradual symptom regulation and the reinforcement of adaptive coping processes.

Participants maintained a high compliance rate of 80% over the 8 weeks, consistently adhering to the EMA/EMI protocol. Most participants completed 2 to 3 EMA/EMI per day throughout the study, demonstrating stable usage patterns without sharp declines at any time point. This suggests that EMA/EMI use was naturally integrated into the participants’ daily routines. The provision of weekly personalized feedback reports might have further supported sustained engagement by reinforcing a sense of individualized care and ongoing monitoring. Given that user attrition has been identified as a major challenge in digital mental health intervention research, the stable engagement patterns observed in this study demonstrate that the EMA/EMI approach can be an acceptable and sustainable intervention modality for young adults. These improvements are broadly consistent with those obtained from digital therapeutics and app-based interventions that reported reductions in anxiety and depressive symptoms [71,72].

At the same time, although the frequent EMA prompts and repeated self-reporting were generally well tolerated, they were perceived as cognitively or emotionally demanding in certain situations. In particular, during periods of heightened stress, users’ receptivity to intensive assessment may vary, highlighting the importance of flexibly balancing assessment frequency with user burden in the design of EMA/EMI systems. The streamlined nature of the EMA/EMI tasks might have contributed to the high levels of engagement observed in this study. However, the therapeutic effects demonstrated in this population with mild-to-moderate anxiety symptoms may not readily generalize to individuals with chronic anxiety or more severe psychiatric conditions, for whom more intensive or clinically supervised interventions may be necessary.

Significant changes in several physiological and behavioral indicators were also observed in the digital phenotype analysis. Deep sleep duration increased significantly while light sleep decreased, and GPS-based movement distance decreased significantly. In addition, the proportion of measurements falling within the low-heart-rate range increased significantly over time, which is to some extent consistent with lower physiological arousal. However, heart rate alone is a weak index of relaxation. HRV, which reflects parasympathetic modulation, would be a more informative physiological marker. Furthermore, these analyses were not adjusted for physical activity. A significant correlation was identified between PHQ-9 and deep sleep duration, indicating a possible association between depressive symptoms and deep sleep. This interpretation assumes reliable wearable sleep-stage detection and, given the small sample, requires cautious reading.

The concurrent increase in deep sleep and reduction in light sleep may indicate an overall improvement in sleep quality, which has been associated with reduced physiological arousal and greater emotional stability. Similarly, the increasing proportion of low heart rate measurements may reflect a more stable pattern of autonomic regulation, consistent with reduced sustained stress or anxiety-related arousal in daily life. Naturalistic studies combining wearable sensing with EMA have similarly examined the physiological correlates of affect and stress in everyday settings, albeit with varying predictive accuracy [42,54,73]. Although the decrease in movement distance may appear negative at first glance, when considered alongside the increasing trend in step count, it suggests that regular activity patterns within primary living areas were established rather than more extensive movement.

The interview analysis yielded 5 major themes in participants’ experiences with the intervention: enhanced self-awareness, promotion of data-driven reflection, support for behavioral change, maintenance of intrinsic motivation, and expansion of active treatment. These themes suggest that the integration of EMA/EMI systems with digital phenotype data contributed to meaningful changes in clinical outcomes, behavioral patterns, and participants’ capacity for self-management.

The combination of real-time self-reported data and objective physiological indicators helped participants develop a more coherent understanding of their emotional and physical states and appears to have been particularly beneficial for individuals with elevated anxiety, who often experience discrepancies between their physical symptoms and subjective perceptions. By providing objective reference points alongside subjective assessments, the system helped participants recognize patterns that might otherwise have remained unnoticed and supported the development of more targeted self-management strategies. Furthermore, the finding that participants independently explored additional therapeutic modules beyond the core app functions suggests that the intervention successfully fostered user autonomy. The therapeutic benefits were thus not confined to moments of active app use but extended into participants’ daily lives through the self-initiated exploration and application of learned strategies.

### Design Implications

#### Overview

The EMA/EMI-based intervention in this study was associated with young adults’ engagement- and intervention-related outcomes in real-world contexts. Because the design did not allow the comparison of the conditions with and without wearable data, the added value of the wearable component cannot be isolated and has thus been discussed only in terms of user experience. Three major design implications were identified to inform the development and integration of automated EMA/EMI-based interventions for mental health care.

#### Temporal Calibration for Personalized Intervention Delivery

An initial adjustment and calibration phase is needed to account for individual differences in emotional expression and the interpretation of EMA response scales. Participants reported that the repeated EMA responses facilitated a clearer recognition of their emotional and behavioral patterns by encouraging reflection on internal states that they might otherwise not have noticed.

During early use, participants experienced uncertainty in translating subjective emotional intensity into numerical scores. Rather than relying on predefined standards, users gradually developed personalized reference points through repeated interaction with the EMA system. Over time, EMA scoring became aligned with individual emotional experiences, indicating that score interpretation is shaped through use rather than established at baseline.

These findings underscore the importance of incorporating an explicit calibration phase during early system use. By jointly analyzing EMA response patterns and wearable-derived digital phenotype data during this phase, individualized emotional baselines can be established that enable the system to identify deviations from users’ typical daily emotional rhythms, allowing personalized interventions to be delivered at moments when support is most needed.

#### Modality Flexibility in Intervention Design

Participants expressed preferences for a variety of intervention formats depending on their situational context and highlighted the importance of flexibility in intervention delivery. The feedback reports supported their reflection on emotional patterns and symptom fluctuations, while the ability to select from among several intervention formats enabled users to engage with the system in ways that aligned with their daily routines.

Participants also emphasized that the feasibility of intervention formats varied across contexts, with some approaches being perceived as difficult to engage with during ongoing activities. This indicates that the effectiveness of the intervention is closely linked not only to its content but also to users’ ability to choose formats that fit their immediate circumstances.

These findings suggest that flexible intervention options enhance users’ sense of choice and self-directed engagement, which may contribute to sustained use and therapeutic effectiveness. Accordingly, EMA/EMI systems should support multiple intervention modalities to accommodate situational constraints, as modality flexibility directly affects users’ ability to engage with interventions at the moment they are delivered.

#### Digital Phenotype–Driven Predictive Emotional Support

The participants in this study reported that reviewing their wearable-derived digital phenotype indicators supported self-reflection on emotional fluctuations and facilitated adjustments in their daily behavior. Notably, the impact of digital phenotype data was enhanced when numerical indicators were accompanied by empathic messages based on their current states.

These findings highlight the importance of an integrated design approach in which predictive interventions informed by digital phenotype data are combined with empathic emotional support delivered through EMA/EMI systems. Rather than relying solely upon reactive interventions provided after explicit user input, this approach emphasizes proactive interventions that synthesize actively self-reported data and passively collected digital phenotypes to anticipate and respond to changes in users’ emotional states. This proactive, context-sensitive direction aligns with the Just-in-Time Adaptive Intervention (JITAI) framework, which we consider a goal for future development rather than a feature of the present system [32].

In this context, digital phenotype data not only function as stand-alone signals for predictive intervention but become meaningful when coupled with empathic emotional support, which reinforces users’ perceptions of being understood and supported by the system.

The participants’ qualitative accounts suggest tentative design considerations to support automated interventions that integrate EMA/EMI systems with wearable-derived digital phenotypes, including adaptive calibration, flexible intervention delivery, and the use of digital phenotype data for predictive intervention combined with empathic emotional support, to facilitate context-responsive mental health care.

By leveraging multidimensional data to detect meaningful changes in users’ emotional states and deliver personalized interventions, EMA/EMI-based approaches can closely align with users’ lived experiences. At the same time, the design of such systems should prioritize the secure handling of sensitive biometric data and the protection of user privacy as fundamental principles to ensure user-centered and sustained implementation.

### Limitations

This study has several limitations that should be borne in mind. First, the exploratory single-arm design limits causal inference regarding the observed changes. Without a control group, natural course, regression to the mean, recruitment bias, and expectancy effects cannot be excluded. In addition, because participants could optionally use other therapeutic modules within ANZEILAX-Green, the observed changes cannot be attributed specifically to the EMA/EMI or wearable components.

Second, the wearable-derived indicators were treated as contextual physiological and behavioral signals rather than validated objective measures of anxiety. Therefore, these findings should be interpreted cautiously. Repeated self-monitoring may also have affected participants differently, as a small number of participants reported heightened awareness of anxiety during periods of elevated distress. Future EMA-based interventions should therefore consider individual differences in response to repeated self-assessment and balance monitoring intensity with user burden [74].

Third, although the sample size was sufficient for this exploratory assessment of feasibility and acceptability, it limited the analysis of individual differences and the generalizability of the findings. In addition, because no follow-up assessment was conducted, it remains unknown whether the observed improvements were sustained beyond the intervention period. Future studies should use larger and more diverse samples and include longer follow-up periods to examine the durability of the observed effects.

### Conclusions

In conclusion, this study provides preliminary evidence for the feasibility, acceptability, and potential clinical benefits of ANZEILAX-Green, an EMA/EMI-based mobile intervention, in reducing anxiety symptoms among young adults. The integration of EMA/EMI systems with wearable-derived digital phenotype data enables a comprehensive assessment of subjective symptom changes and objective indicators, including physiological and behavioral data, demonstrating how personalized feedback can operate within everyday contexts. This integrative approach extends the clinical applicability of mobile mental health interventions by allowing the multidimensional emotional changes associated with anxiety symptoms in daily life to be captured. A randomized controlled trial is thus needed to confirm its effectiveness.

Beyond symptom reduction, the findings suggest that EMA/EMI-based interventions integrated with digital phenotyping can support ongoing self-management by mitigating symptom exacerbation in everyday contexts and facilitating the application of therapeutic strategies outside moments of active app use. In addition, the observed clinical effects appear to be supported by an integrated feedback structure that leverages digital phenotype data to enhance the acceptability and enactment of the intervention in daily life.

Furthermore, the findings provide empirical support for the design and advancement of automated digital mental health interventions that reflect individuals’ ongoing states and contextual conditions in the feedback they provide. By showing that the integration of EMA/EMI systems with digital phenotyping extends and supports self-awareness, reflection, and self-management, this study highlights a scalable and clinically meaningful direction for future context-responsive digital mental health interventions.

## Abbreviations

ACT: acceptance and commitment therapy
BAI: Beck Anxiety Inventory
DTx: digital therapeutics
EMA: ecological momentary assessment
EMA/EMI: ecological momentary assessment and ecological momentary intervention
EMI: ecological momentary intervention
GAD-7: Generalized Anxiety Disorder 7-item scale
HRV: heart rate variability
I-PANAS-SF: International Positive and Negative Affect Schedule Short Form
JITAI: Just-in-Time Adaptive Intervention
MAUQ: mHealth App Usability Questionnaire
OLS: ordinary least squares
PHQ-9: Patient Health Questionnaire-9
RSES: Rosenberg Self-Esteem Scale

## Data Availability

The datasets generated or analyzed during this study are not publicly available due to participant confidentiality. A simplified, deidentified dataset can be made available from the corresponding author upon reasonable request.
